# Placenta Prævia

**Published:** 1856-02

**Authors:** 


*Placenta Prœvia.* — Mr. J. Thomson gives (*Glasgow Medical Journal*, July, 1855) a statistical report of three thousand three hundred cases of obstetricy, in which there were five cases of placental presentation. The mother, in three of these cases, recovered; in two died; all the children were stillborn. The details of two of these cases which he gives are as follows:

CASE 1. "I was sent for to visit Mrs. C., resident four miles from town, on the 11th October, at four P. M., and who, I was informed, had experienced repeated attacks of hemorrhage during the preceding two months. On arriving at her house, I found that she had already fainted several times, and that with every successive pain, the flooding was severe. Her skin was cold to the touch, the pulse rapid and scarcely perceptible at the wrist. On examination, the placenta was felt protruding through the soft and yielding os uteri.

Without loss of time, the hand was introduced, the child turned, and delivered stillborn. Immediately afterwards, the placenta came away, almost without any further loss of blood. All this was accomplished in less than fifteen minutes after I entered the house. I remained with the patient for an hour afterwards, and during this time there was no tendency to syncope, although the pulse still remained extremely weak, and beating upwards of 120. After giving suitable instructions, I left, requesting that I should be sent for if she became worse during the night. Next day I found her weak, but without any other complaint. The pulse was somewhat stronger, though nearly as rapid as before. From this period she continued slowly to improve up to the eighth day, when I was again summoned in great haste. I found that, while sitting up in bed, taking breakfast, she had fainted. She had been immediately placed in the horizontal position, and had the pillows removed from under her head. This, and the administration of some cordials, soon restored her to consciousness. From this time she continued to improve, till the beginning of the following week. She then began to talk incoherently, and to show decided indications of an attack of puerperal mania."

Case 2. "At eight, A.M. of the 1st December, I was sent for to visit Mrs. A., who was said to be advanced to her eighth month of pregnancy. About three weeks previous to this period, she had become affected with a severe cough, which often prevented her from enjoying rest during the night. About an hour before my visit, and while suffering from a severe paroxysm of coughing, a sudden discharge of blood took place from the vagina. When I arrived, I found that the flooding still continued, and that occasional pains were felt in the back. When the large coagula which plugged the vagina had been cleared away, the os uteri was felt high up, and completely closed. In these circumstances, I followed the advice given by Burns, when he says: 'If the os uteri be firm and close in a first attack, we ought to use the plug, which will restrain the hemorrhage, and insure the present safety of the patient.' This measure arrested the hemorrhage during the whole day. Next morning, about eight o'clock, I was again called. On inquiry, I found that the plug had been removed during the night, and that the flooding had been immediately renewed. Through some mistake, I was, unfortunately, not sent for some hours afterwards. Though the flooding had evidently been great, yet the patient had shown no tendency to syncope, but looked pale and ex·sanguine, and the pulse had raised to 150. The os uteri was now found dilated to the size of half-a-crown, and the placenta entirely implanted over it. This being ascertained, the turning and delivery of the child was the work of a very few minutes, and this was effected with very little additional loss of blood. The patient expressed herself as comfortable, and with the exception of the rapidity of the pulse, everything seemed to wear a favorable aspect. There was no jactitation, no tendency to syncope, and nothing to excite immediate alarm, but the extent of the flooding, which had evidently been great. The placenta was brought away very soon after the birth of the child, and the uterus above the pubis was felt to be firm and well contracted. A glass of wine was given, and this was succeeded by a dose of morphia. During the day she continued in a pretty comfortable state, and was able to take some light nourishment. By the morning of the third, reäction had taken place, the skin had become hot, the breathing hurried, the thirst urgent, the tongue dry, and the pulse had risen to 160. A diaphoretic and sedative mixture was now prescribed. By the evening she was slightly delirious, restless, and uneasy. The uterus from the beginning was felt firmly contracted, and the abdomen was soft, without tumidity, or pain even on firm pressure. The lochial discharge was moderate from the first. On the morning of the fourth, the heat of the skin was reduced to a moderate temperature, but the pulse was not improved. The condition of the patient was now perilous in the extreme, and every appliance which science or experience could suggest was unremittingly and perseveringly applied. All, however, was of no avail, and she died on the morning of the fifth, exactly three days after her delivery."

